# BiGATAE: a bipartite graph attention auto-encoder enhancing spatial domain identification from single-slice to multi-slices

**DOI:** 10.1093/bib/bbae045

**Published:** 2024-02-21

**Authors:** Yuhao Tao, Xiaoang Sun, Fei Wang

**Affiliations:** Shanghai Key Lab of Intelligent Information Processing, Handan Street, 200433 Shanghai, China; School of Computer Science and Technology, Fudan University Handan Street, 200433 Shanghai, China; Shanghai Key Lab of Intelligent Information Processing, Handan Street, 200433 Shanghai, China; School of Computer Science and Technology, Fudan University Handan Street, 200433 Shanghai, China; Shanghai Key Lab of Intelligent Information Processing, Handan Street, 200433 Shanghai, China; School of Computer Science and Technology, Fudan University Handan Street, 200433 Shanghai, China

**Keywords:** spatial transcriptomics, identification of spatial domains, bipartite graph attention network, multiple slices, seamlessly integrate

## Abstract

Recent advancements in spatial transcriptomics technology have revolutionized our ability to comprehensively characterize gene expression patterns within the tissue microenvironment, enabling us to grasp their functional significance in a spatial context. One key field of research in spatial transcriptomics is the identification of spatial domains, which refers to distinct regions within the tissue where specific gene expression patterns are observed. Diverse methodologies have been proposed, each with its unique characteristics. As the availability of spatial transcriptomics data continues to expand, there is a growing need for methods that can integrate information from multiple slices to discover spatial domains. To extend the applicability of existing single-slice analysis methods to multi-slice clustering, we introduce BiGATAE (Bipartite Graph Attention Auto Encoder) that leverages gene expression information from adjacent tissue slices to enhance spatial transcriptomics data. BiGATAE comprises two steps: aligning slices to generate an adjacency matrix for different spots in consecutive slices and constructing a bipartite graph. Subsequently, it utilizes a graph attention network to integrate information across different slices. Then it can seamlessly integrate with pre-existing techniques. To evaluate the performance of BiGATAE, we conducted benchmarking analyses on three different datasets. The experimental results demonstrate that for existing single-slice clustering methods, the integration of BiGATAE significantly enhances their performance. Moreover, single-slice clustering methods integrated with BiGATAE outperform methods specifically designed for multi-slice integration. These results underscore the proficiency of BiGATAE in facilitating information transfer across multiple slices and its capacity to broaden the applicability and sustainability of pre-existing methods.

## INTRODUCTION

Recent years have seen significant progress in spatial transcriptomics technologies [1]. These advancements offer dual functionality, enabling the simultaneous extraction of spatial information and gene expression abundance from tissue samples [2]. This innovation empowers researchers to explore cellular diversity in terms of spatial arrangement and functional characteristics. Spatial transcriptomics technologies are pivotal in revolutionizing our understanding of the molecular basis of diseases [3] and hold great promise for advancing diagnostic and therapeutic strategies [4].

Clustering is crucial in spatial transcriptomics data analysis [5], serving as a fundamental tool for dissecting the intricate spatial organization of gene expression patterns within tissues [6]. By grouping cells with similar transcriptional profiles into distinct clusters, researchers can unveil hidden spatial relationships and heterogeneity in cellular populations [7]. This process aids in identifying different cell types, their spatial distribution, and unique cell subpopulations with specific functions [8, 9]. Clustering also aids in the recognition of spatially co-expressed genes, revealing potential regulatory networks or co-functional groups of genes. Additionally, clustering facilitates the comparison of different tissue samples or conditions, enabling researchers to discern how gene expression patterns vary across space and in response to various stimuli or disease states [10]. This knowledge is invaluable in understanding the underlying mechanisms of diseases and can guide the development of more precise diagnostic and therapeutic strategies [11].

In the realm of spatial transcriptomic clustering, various methods have emerged, each employing distinct strategies to decipher the intricate interplay of gene expression within tissue structures. These methods are categorized into four primary groups based on their underlying techniques. The first category encompasses foundational techniques such as K-means and Louvain clustering [12], focusing solely on gene expression profiles to delineate cellular regions, similar to clustering in single-cell transcriptome data. While these methods are straightforward, their application lacks spatial information guidance, potentially resulting in an inadequate representation of cellular spatial correlations within tissue structures. The second category integrates spatial location data into traditional methods, with notable approaches like Giotto [13], BayesSpace [14] and SC-MEB [15] enhancing clustering accuracy. However, these methods may struggle to fully discern the complexity of spatial interactions, particularly given the high dimensionality of gene expression data. The third category employs deep learning techniques, as seen in MUSE [16], TIST [17] and stLearn [18], proficiently interpreting the complex non-linear correlations present in high-dimensional gene expression data. Nevertheless, their integration of spatial information is not exhaustive, leading to the formation of the fourth category. This category harnesses the advanced capabilities of Graph Neural Network (GNN) models, standing at the forefront of spatial clustering methods, specifically designed for analyzing spatial transcriptomic data. Prominent methodologies within this cadre include SpaGCN [19], SEDR [20], CCST [21] and STAGATE [22]. GNNs distinguish themselves in the analysis of spatial transcriptomic data by conceptualizing each spot as a discrete node within the network, guided by locational data that define the relational schema between spots, ensuring congruence with authentic biological scenarios.

With advancing technology, there are more and more multiple consecutive spatial transcriptomic slices. These consecutive slices exhibit strong spatial similarity, with information transmission between adjacent slices enhancing clustering effectiveness. Though some integrated analysis tools for multiple slices, such as STAligner [23], GraphST [24], PRECAST [25] have emerged, these methods are primarily designed for integrating multiple similar or dissimilar slices, addressing issues like batch effects. They do not specifically consider leveraging the adjacency information between consecutive slices to improve the precision of cell identification and discovery.

Considering the myriad distinctions between these methods, each possessing its unique design and specialized problem-solving capabilities, there is no universally optimal method that can be applied across all scenarios. The efficacy of these methods fluctuates based on several factors, including the technology employed, the intricacy of the profiled tissue, the specific biological queries at hand, the computational resources available and ease of implementation.

In this paper, we aim to fully leverage a variety of existing clustering tools designed for individual spatial transcriptomic slices and extend their application to the clustering analysis of multiple consecutive slices. To accomplish this, we introduce BiGATAE, a tool akin to a shell. It can be seamlessly integrated with existing single-slice clustering methods, extending their applicability to multi-slice clustering, with the goal of enhancing single-slice clustering by incorporating information from neighboring slices. We have chosen several representative methods, and experimental results across multiple datasets demonstrate that the incorporation of BiGATAE leads to improved clustering outcomes, affirming its effective transmission of information between adjacent slices. Moreover, when compared to existing multi-slice analysis methods, our extended single-slice approach, empowered by BiGATAE, achieves superior clustering results.

## METHODS

### Overview of BiGATAE

**Figure 1:**
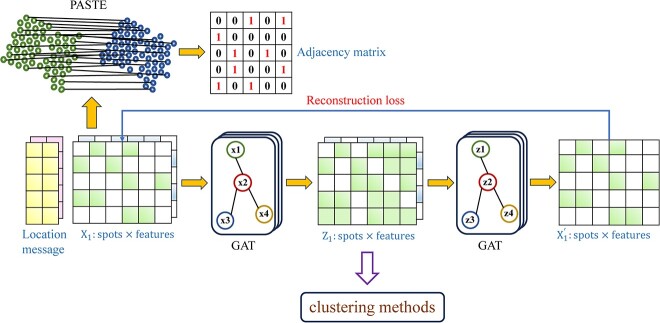
Framework of the BiGATAE method.

The BiGATAE framework, as illustrated in Figure 1, comprises two main steps. Firstly, it establishes the alignment of neighboring slices, and secondly, it facilitates the transfer of information from adjacent slices to improve the clustering performance of the target slice. More specifically, BiGATAE employs the PASTE algorithm [26] to identify correspondences between spots in adjacent slices, leading to the creation of an adjacency matrix for spots in neighboring slices and the construction of a bipartite graph. Subsequently, BiGATAE introduces graph neural networks to facilitate information transfer, akin to an encoder-decoder mechanism. The encoder employs a graph attention layer to aggregate gene expression data from neighboring slices through the bipartite graph and into the target slice. Conversely, the decoder utilizes another graph attention layer to eliminate gene expression information from neighboring slices in the target slice, effectively reversing the process of the encoder. By following this two-step procedure, BiGATAE effectively enhances gene expression in the target slices by leveraging information from adjacent slices. The resulting enhanced expression matrix offers a comprehensive and refined representation of gene expression patterns within the target slice that can seamlessly integrate with existing clustering methods.

### Data preprocessing

BiGATAE takes spatial gene expression data and positional information from two adjacent slices as input. In all datasets, we carefully select 3000 highly variable genes and apply log transformation and normalization to the gene expression data using scanpy [27].

Following these preprocessing steps, each slice can be represented as a set of matrices ($E_{N \times D}$, $S$), where $E_{N \times D}$ denotes the processed matrices that are contained in an N$\times $D matrix with N spots and D features, and $S$ represents the two-dimensional spatial coordinates of each spot. $E_{i}$ denotes the gene expression profile of the spot $i$.

### Construction of a bipartite graph by PASTE alignment method

BiGATAE employs the PASTE alignment method to establish a bipartite graph connecting the target slice with its neighboring slice. PASTE generates an alignment matrix based on optimal transmission theory. This matrix is probabilistic in nature, with each element indicating the likelihood that a spot in one slice is adjacent to a spot in another slice within the three-dimensional tissue. A spot within one slice may be associated with several spots in the other slice. This probabilistic matrix is then converted into a binary adjacency matrix to construct a bipartite graph denoted as $BG(U, V,\varepsilon )$. Here, $U$ represents the node set of the target slice, $V$ represents the node set of adjacent slices, $u_{i}$ denotes the $i$th node in set $U$, and $v_{i}$ signifies the $i$th node in set $V$. The symbol $\varepsilon $ represents the edges of the bipartite graph.

### Bipartite attention autoencoder

BiGATAE introduces a bipartite attention autoencoder to aggregate information from adjacent slices. The autoencoder primarily comprises two key components: an encoder module designed to incorporate information from adjacent slices and a decoder module to reconstruct the target slice’s original expression. Both modules utilize a single attention layer within the bipartite graph framework, inspired by Graph Attention Network (GAT) [28]. GAT is a graph neural network model that leverages graph structure and node features to improve representation learning. It employs a learnable weight matrix for node representation transformation and an attention mechanism to assess the importance of neighboring nodes for each target node. The attention coefficients facilitate the aggregation of neighboring node representations, enhancing the target node’s representation. The GAT framework comprises two main components: attention coefficient calculation and node updates, as outlined below: 


(1) \begin{align*} & \alpha_{ij} = \frac{\exp \left( \mathrm{LeakyReLU} \left( a^{\top} [W h_{i} || W h_{j}] \right) \right)}{\sum_{k \in N_{i}} \exp \left( \mathrm{LeakyReLU} \left( a^{\top} [W h_{i} || W h_{k}] \right) \right)} \end{align*}

(2) \begin{align*} & h_{i} = \sigma \left( \sum_{j \in N_{i}} \alpha_{ij} W h_{j} \right) \end{align*}

where $\alpha _{ij}$ denotes the attention coefficient between node $i$ and node $j$, $a$ is a learnable weight vector within the attention mechanism, measuring the similarity between nodes. Here, $h_{i}$ stands for the representation of node $i$, and $h_{j}$ signifies the representation of its neighbor, node $j$. The weight matrix $W$ is employed to transform node representation into a new space. Additionally, $\sigma $ and $\mathrm{LeakyReLU}$ serve as activation functions that enhance the model’s nonlinearity. Furthermore, the symbol || denotes the vector concatenation operation, which combines two vectors into a new composite vector.

The attention layer in the bipartite graph design draws inspiration from GAT, and the adapted formula for the encoder module is as follows: 


(3) \begin{align*}& \alpha_{u_{i},v_{j}} = \frac{\exp \left( \mathrm{LeakyReLU} \left( a^{\top} [E_{u_{i}} || E_{v_{j}} \right) \right)}{\sum_{v_{k} \in N^{\varepsilon}_{u_{i}}} \exp \left( \mathrm{LeakyReLU} \left( a^{\top} [E_{u_{i}} || E_{v_{k}}] \right) \right)}\end{align*}

As the goal of the encoder module is to gather information from adjacent slices, rather than the slice itself, we have eliminated the W matrix and substituted $h_{i}$ and $h_{j}$ with $E_{u_{i}}$ and $E_{v_{j}}$ in Formula 3, respectively. Furthermore, we define $bga_{\varepsilon }(u_{i})$ as the encapsulation of information from the adjacent slice through the bipartite graph in Formula 4. The encoder process is then outlined in Formula 5: 


(4) \begin{align*} & bga_{\varepsilon}(u_{i}) = ReLU\left( \sum_{v_{k} \in N^{\varepsilon}_{u_{i}}} \alpha_{u_{i},v_{k}} E_{v_{k}} \right) \end{align*}

(5) \begin{align*} & z_{u_{i}} = E_{u_{i}} + bga_{\varepsilon}(u_{i}) \end{align*}

where $z_{u_{i}}$ denotes an enhanced matrix resulting from the encoder process. It subsequently serves as the input for pre-existing clustering methods.

The decoder process outlined in Formula 8 is to eliminate information from adjacent slices in the enhanced representation $z_{u_{i}}$ to reconstruct the original representation of the target slice: 


(6) \begin{align*} & \overline{\alpha}_{u_{i},v_{j}} = \frac{\exp \left( \mathrm{LeakyReLU} \left( a^{\top} [z_{u_{i}} || E_{v_{j}} \right) \right)}{\sum_{v_{k} \in N^{\varepsilon}_{u_{i}}} \exp \left( \mathrm{LeakyReLU} \left( a^{\top} [z_{u_{i}} || E_{v_{k}}] \right) \right)} \end{align*}

(7) \begin{align*} & \overline{bga}_{\varepsilon}(u_{i})=ReLU\left( \sum_{v_{k} \in N^{\varepsilon}_{u_{i}}} \overline{\alpha}_{u_{i},v_{k}} E_{v_{k}} \right) \end{align*}

(8) \begin{align*} & \overline{E}_{u_{i}} = z_{u_{i}} - \overline{bga}_{\varepsilon}(u_{i}) \end{align*}

where $\overline{E}_{u_{i}}$ represents the reconstructed the matrix of $E_{u_{i}}$ from the decoder module.

### Loss function

The loss function of BiGATAE is the mean square error: 


(9) \begin{align*}& MSE = \frac{1}{N}\sum_{i=1}^{N}(E_{u_{i}} - \overline{E}_{u_{i}})^{2}\end{align*}

## RESULT

To assess the effectiveness of BiGATAE, we carefully selected a range of representative clustering methods for single-layer slice analysis, including Louvain, Leiden, stLearn, SpaGCN, SEDR and STAGATE. Employing these chosen methods, we integrated the BiGATAE technique to enable the exchange of information between diverse slices. We conducted comparative analyses, assessing the clustering performance of each method both before and after the incorporation of BiGATAE. Additionally, we compared the clustering performance of GraphST and STAlinger, which are multi-layer slice integration methods, with the single-layer slice approach bolstered by BiGATAE. These two comparative aspects serve to demonstrate the efficacy and impact of BiGATAE in enhancing clustering analyses.

### Baselines

#### Louvain and Leiden

The Louvain [29] and Leiden [30] algorithms are prominent in community detection for networks. Louvain optimizes modularity, revealing community structures by iteratively adjusting node assignments. Leiden improves upon Louvain by introducing a refinement step, enhancing solution stability and robustness, especially in complex or noisy networks.

#### stLearn

stLearn [18] concurrently integrates gene expression data, spatial coordinates and histological information. It employs a pre-trained convolutional neural network to construct a histological similarity graph and conducts a two-step spatial clustering procedure. In the initial step, either Louvain or k-means clustering is applied to the graph’s adjacency matrix, considering both histological similarity and expression abundance. Subsequently, in the second step, spatial information is utilized to identify sub-clusters.

#### SpaGCN

SpaGCN [19] leverages average RGB channel color information in histological images, coupled with two-dimensional spatial coordinates, as feature data. These features drive the computation of spot similarities, resulting in a weighted undirected graph. SpaGCN further utilizes a graph convolutional network to transfer gene expression information between neighboring spots and employs iterative clustering to accurately determine spatial domains.

#### SEDR

SEDR [20] first uses an autoencoder with two fully connected layers to learn a low-dimensional latent representation of the gene expression matrix, and then embeds it together with spatial information through a variational graph autoencoder. Finally, the decoder of SEDR reconstructs the fused low-dimensional latent representation into the original gene expression matrix.

#### STAGATE

Built upon the graph attention autoencoder framework, STAGATE [22] adaptively learns low-dimensional features by integrating transcriptomic and spatial information. The approach involves employing Iterative Closest Point to pre-align multiple slices and manually refining slice alignment to achieve a three-dimensional spatial domain.

#### GraphST

GraphST [24] represents a comprehensive solution featuring three distinct modules designed to address specific tasks in the realm of graph self-supervised contrastive learning. The first module focuses on spatially informed clustering, leveraging graph-based techniques to identify clusters within complex datasets. The second module addresses the integration of multiple tissue sections in both vertical and horizontal orientations, providing a holistic understanding of spatial relationships. The third module enables spatially informed cell type deconvolution by projecting single-cell RNA sequencing data onto spatial transcriptomics data.

#### STAligner

STAligner [23] facilitates the integration and alignment of spatial transcriptomics (ST) datasets by utilizing both gene expression and spatial coordinate matrices from multi-layer ST slices. It comprises two key components. The first is a graph attention autoencoder to learn and understand spatial relationships between different spots in a slice. The second component is a point triplet learning module that is specifically developed for batch correction. This component ensures that the data are properly aligned and corrected.

### Validation datasets

We have curated three comprehensive spatial transcriptome datasets available on public platforms, extensively utilized for testing various spatial clustering algorithms. The first dataset is sourced from the human dorsolateral prefrontal cortex [31], comprising 12 tissue slices. These slices are divided into three groups, each consisting of four consecutive slices from the same tissue, with designated identifiers: 151507–151510, 151669–151672 and 151673–151676. The second dataset encompasses spatial transcriptome data from the breast cancer dataset [32], featuring four consecutive adjacent tissue sections from a single breast cancer tissue. The third dataset originates from the human skin squamous cell carcinoma dataset [33], comprising skin squamous cell carcinoma tissues from five patients. Four of these patients provided three adjacent consecutive slices, while the remaining two patients contributed two adjacent consecutive slices each. Details of spot numbers in each slice across the three datasets are presented in Table 1. It is noteworthy that the first dataset has known cell-type annotations, while the last two datasets have not.

**Table 1:** The information from three datasets used for experimental evaluation

Human dorsolateral prefrontal cortex dataset		Breast cancer dataset		Human skin squamous cell carcinoma dataset		
Slice number	Number of spots	Slice number	Number of spots	Patient number	Slice number	Number of spots
151508	4384	1	254	Patient2	P2_ST_rep1	666
151509	4789	2	250		P2_ST_rep2	646
151510	4634	3	263		P2_ST_rep3	638
151669	3661	4	262	Patient4	P4_rep1	744
151670	3498				P4_rep2	696
151671	4110			Patient5	P5_ST_rep1	590
151672	4015				P5_ST_rep2	521
151573	3639				P5_ST_rep3	521
151574	3673			Patient6	P6_rep1	3650
151575	3592				P6_rep2	3838
151576	3460			Patient9	P9_ST_rep1	1145
					P9_ST_rep2	1071
					P9_ST_rep3	1182
				Patient10	P10_ST_rep3	608
					P10_ST_rep2	621
					P10_ST_rep3	462

### Evaluation metrics

We employed various evaluation metrics to assess the performance in spatial domain identification. When cell type annotations are available, we utilized five clustering evaluation metrics [34]: Adjusted Rand Index (ARI), Adjusted Mutual Information (AMI), Normalized Mutual Information (NMI), Homogeneity Score (Homo) and Completeness Score (Comp). When cell type annotations are unknown, we employed two clustering evaluation indices: Silhouette Coefficient (SC) and Calinski–Harabasz Index (CHI). The formulas for ARI, AMI, NMI, Homo, Comp, CHI and SC are as follows: 


(10) \begin{align*}& ARI = \frac{\sum_{ij}\binom{n_{ij}}{2} - [\sum_{i}\binom{a_{i}}{2}\sum_{j}\binom{b_{j}}{2}]/\binom{n}{2}}{\frac{1}{2}[\sum_{i}\binom{a_{i}}{2}+\sum_{j}\binom{b_{j}}{2}] - [\sum_{i}\binom{a_{i}}{2}\sum_{j}\binom{b_{j}}{2}]/\binom{n}{2}}\end{align*}

where $n$ is the total number of samples, $n_{ij}$ is the number of samples assigned to both class $i$ and class $j$, $a_{i}$ is the number of samples assigned to class $i$, and $b_{j}$ is the number of samples assigned to class $j$: 


(11) \begin{align*} & MI(A, B) = H(A) - H(A|B) \end{align*}

(12) \begin{align*} & NMI = \frac{MI(A, B)}{\sqrt{H(A) \cdot H(B)}} \end{align*}

(13) \begin{align*} & AMI = \frac{MI(A, B) - E(MI(A, B))}{\max(H(A), H(B)) - E(MI(A, B))} \end{align*}

where $MI(A, B)$ represents the mutual information between $A$ and $B$, $H(A)$ and $H(B)$ represent the entropy of $A$ and $B$, respectively, and $E(MI(A, B))$ represents the mutual information expectation under the stochastic model: 


(14) \begin{align*} & Homo = 1 - \frac{H(C|K)}{H(C)} \end{align*}

(15) \begin{align*} & Comp = 1 - \frac{H(K|C)}{H(K)} \end{align*}

where $C$ is the real category labels, $K$ is the clustering results, $H(C)$ is the entropy of $C$, $H(K)$ is the entropy of $K$ and $H(K|C)$ is the entropy of $K$, given $C$: 


(16) \begin{align*}& \textrm{SC}(i) = \frac{b(i) - a(i)}{\max[a(i), b(i)]}\end{align*}

where $a(i)$ is the average distance between sample $i$ and other samples in the same cluster, $b(i)$ is the average distance between sample $i$ and all samples in the nearest other cluster: 


(17) \begin{align*}& \textrm{CHI} = \frac{B(K)}{W(K)} \times \frac{N - K}{K - 1}\end{align*}

where $B(K)$ is the variance between clusters, $W(K)$ is the variance within clusters, $N$ is the total number of samples and $K$ is the number of clusters.

### The integration of BiGATAE significantly enhances the clustering accuracy of various algorithms applied to the human dorsolateral prefrontal cortex dataset

**Figure 2:**
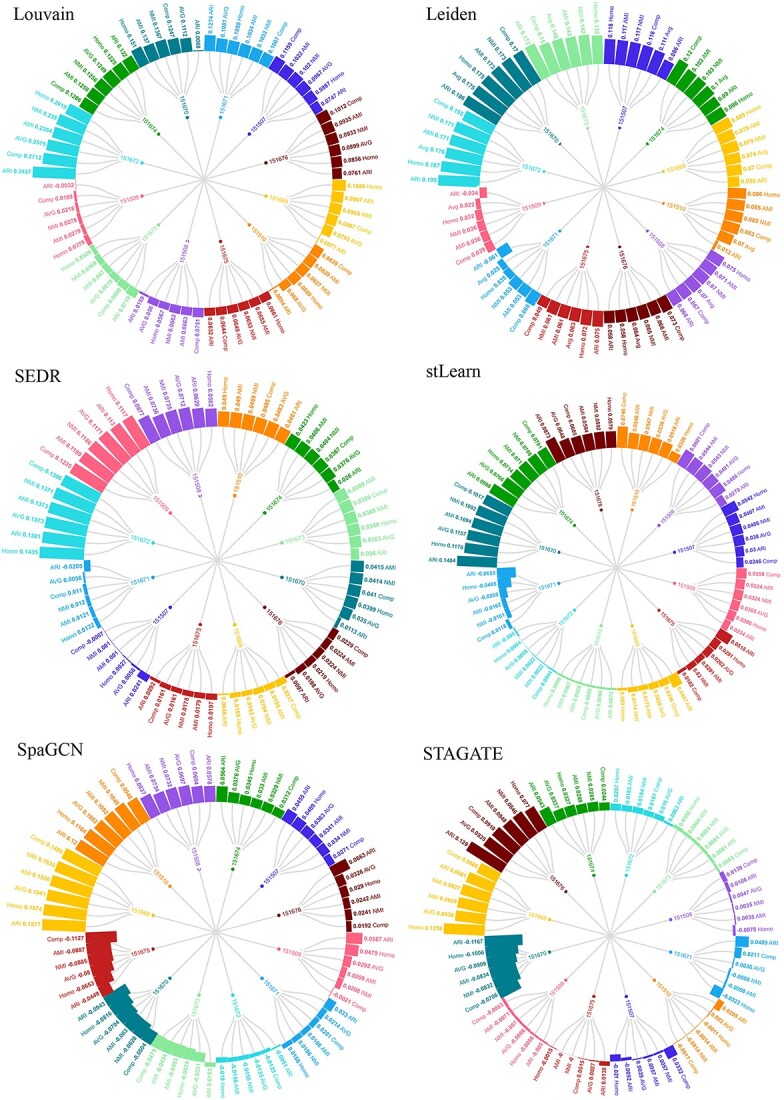
Enhancement of BiGATAE on clustering performance of multiple methods. Each subgraph corresponds to a specific clustering algorithm and presents the changes in performance across 12 slices (151507–151510, 151669–151672 and 151673–151676) before and after integrating BiGATAE. The clustering evaluation metrics examined include ARI, AMI, NMI, Homo, Comp and AVG. In each subgraph, each color represents a distinct slice, and numerical values displayed on the circles indicate positive improvements achieved after integrating BiGATAE, with outward-pointing bars. Conversely, negative values on the circles signify performance declines following the integration of BiGATAE, with inward-pointing bars.

This dataset comprises a total of 12 slices. For Louvain, Leiden, stLearn, SpaGCN, SEDR and STAGATE, we conducted a comparative analysis of clustering performance before and after incorporating the BiGATAE method across all 12 slices. The results are presented in Figure 2. Notably, although STAGATE can be utilized for the integration of multiple slice layers, it necessitates manual alignment of these layers. Therefore, in this study, we have exclusively employed STAGATE for single-layer slice clustering, setting aside its multi-slice integration component.

In Figure 2, upon closer examination of the subgraphs representing the results for Louvain and Leiden, it becomes evident that BiGATAE has a substantial and positive impact on all slices. For Louvain, the enhancement resulting from the integration of BiGATAE can reach as high as 0.34 in ARI, while for Leiden, it can reach up to 0.19 in ARI. These findings unequivocally demonstrate that the integration of adjacent slice information through BiGATAE significantly elevates the clustering performance of both Louvain and Leiden.

Analyzing the results for SEDR reveals a noticeable improvement in clustering performance across nearly all slices. When closely scrutinizing the results for stLearn and STAGATE, an overall performance enhancement is observed across most slices, with only a marginal decline in performance noted for a single slice—specifically, slice 151670 for stLearn and slice 151671 for STAGATE.

For the SpaGCN method, performance sees improvement in 8 out of the 12 slices. The slices with decreased performance in SpaGCN are relatively more numerous in comparison to other methods. This observation may be due to the substantial contribution of information from histological images to the clustering process, which, in turn, offsets the gains from the transfer of expression information across adjacent slices.

In a word, Figure 2 effectively visually conveys the impact of BiGATAE on various spatial clustering methods. It underscores the cases where BiGATAE significantly enhances the results, thereby affirming its effectiveness in improving clustering outcomes across diverse slices.

**Figure 3:**
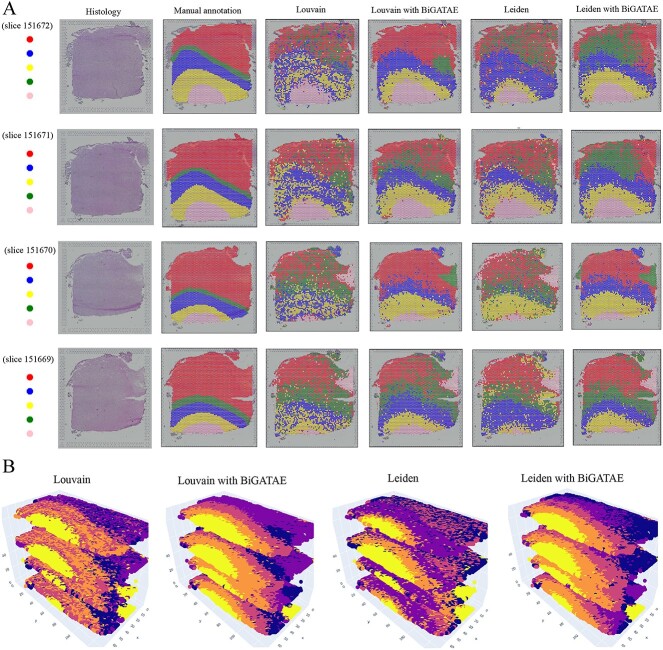
Visualization of spatial domains in 2D and 3D space. **A**, From left to right, the images include histology images, images depicting manually annotated regions, and spatial domains detected by Louvain, Louvain with BiGATAE, Leiden and Leiden with BiGATAE. **B**, Identification of spatial domains across multiple slices.

In order to showcase BiGATAE’s ability to identify spatial domains across multiple slices, we’ve taken slices 151669–151672 as an illustrative example, and the visualization results are presented in Figure 3. Figure 3(A) displays the clustering outcomes for each slice, while Figure 3(B) showcases the 3D spatial domains. Figure 3 highlights that, in addition to the enhancements BiGATAE offers to the Louvain and Leiden algorithms, it also excels at achieving superior 3D spatial clustering results.

**Figure 4:**
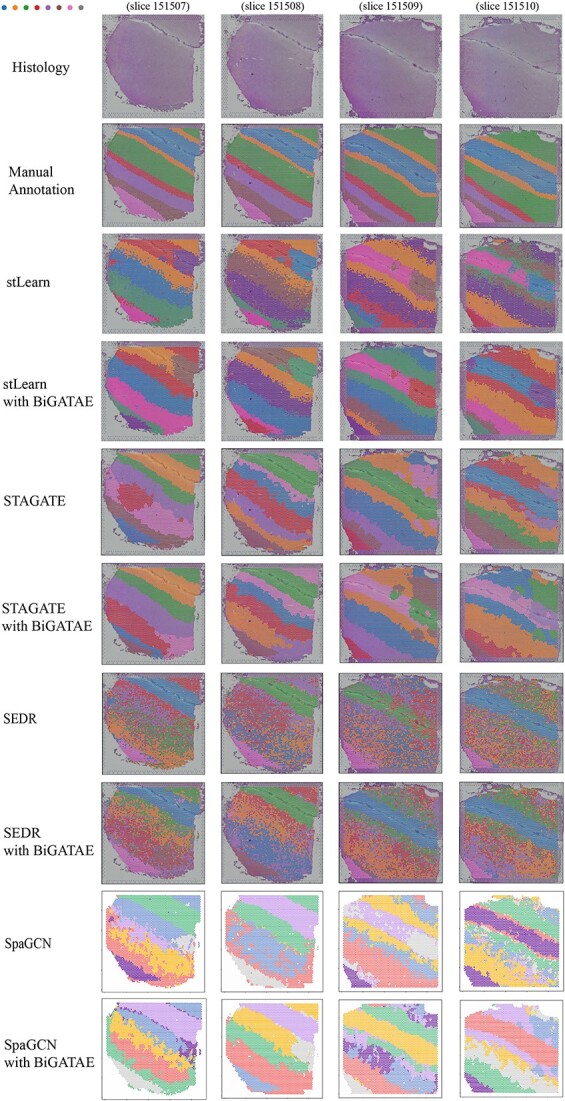
Spatial domains in slices 151507–151510 of human dorsolateral prefrontal cortex data. From top to bottom, the images include histology images, images displaying manually annotated regions, spatial domains detected by stLearn, stLearn with BiGATAE, STAGATE, STAGATE with BiGATAE, SEDR, SEDR with BiGATAE, SpaGCN and SpaGCN with BiGATAE.

To further investigate how BiGATAE facilitates the transmission of information from adjacent slices to enhance the performance of multiple clustering algorithms, we compared visualizations of various clustering methods integrated with BiGATAE, as depicted in Figure 4. Figure 4 vividly illustrates that algorithms incorporating BiGATAE can more accurately delineate cell-type boundaries. In Figure 4, the boundaries of annotated cell types appear relatively smooth, whereas STAGATE, stLearn and SpaGCN display cell type boundaries with more jagged edges, indicating less satisfactory clustering results at these boundaries. However, the integration of BiGATAE significantly smoothens the clustering outcomes at these boundaries, resulting in a notable improvement in clustering performance. STAGATE and SpaGCN are both based on graph neural network methods, constructing graph models based on gene expression profiles and spatial relationships, with each node in the graph corresponding to a cell or spot. The representation of each node aggregates information from neighboring nodes in the graph, forming the basis for clustering. Near the boundaries of cell types, nodes and their neighboring nodes in the graph may not belong to the same cell type, leading to a decrease in clustering performance due to the aggregation of information from different cell types. With the integration of BiGATAE, information from multiple slices implicitly complements the knowledge of cell-type boundaries. This allows a node to better identify neighboring nodes belonging to the same cell type, whether on the same or different slices, enhancing the clarity of information aggregation from neighboring nodes and improving clustering accuracy at cell type boundaries.

**Figure 5:**
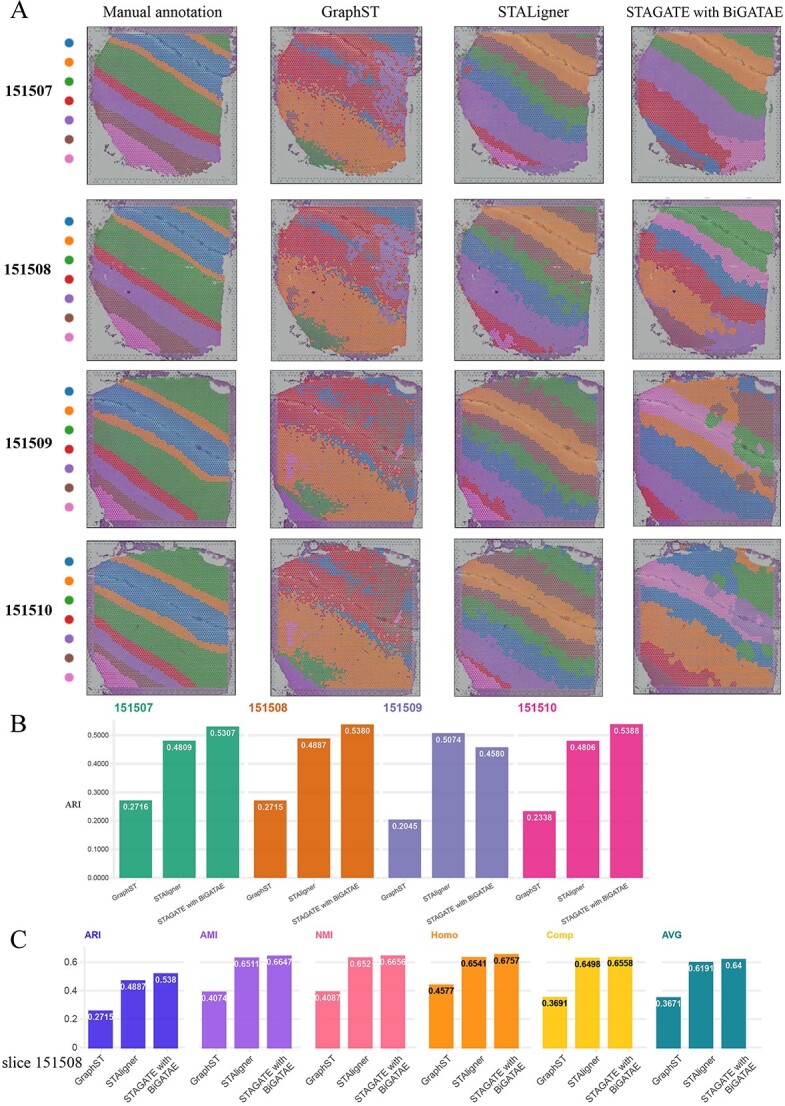
Spatial domains, clustering evaluation metrics in the human dorsolateral prefrontal cortex data (slices 151507–151510). **A**, From left to right, the images include manually annotated images, spatial domains detected by GraphST, STALigner and STAGATE with BiGATAE. **B**, ARI scores for GraphST, STALigner and STAGATE with BiGATAE on four slices (slices 151507–151510). **C**, Results of six clustering evaluation metrics for GraphST, STALigner and STAGATE with BiGATAE on slice 151508.

Lastly, we compared the methods for single-layer slice enhanced with BiGATAE to GraphST and STAligner, both specially designed for multi-slice integration. For single-layer slice clustering, we selected STAGATE, a method developed by Zhang’s group, which is also the creator of STAligner. The comparative results are depicted in Figure 5. It is evident that, in most cases, the clustering performance of STAGATE integrated with BiGATAE surpasses that of STAligner. STAGATE integrating BiGATAE and STAligner exhibit significantly improved clustering performance compared to GraphST, with clustering performance metrics essentially doubling.

In summary, the experimental results above demonstrate that integrating BiGATAE with single-layer slice clustering methods can enhance the performance of each method, with particularly significant improvements for traditional methods like Louvain and Leiden. Furthermore, the integration of BiGATAE with single-layer slice clustering methods outperforms methods originally designed for multi-slice integration. These findings indicate that BiGATAE effectively facilitates the transmission of information between adjacent slices, making it easily adaptable to existing single-layer slice methods and extending their capabilities to multi-slice clustering algorithms.

### The integration of BiGATAE with SEDR yields superior results in the human skin squamous cell carcinoma dataset

To validate the impact of BiGATAE, we also conducted a comparison using the human skin squamous cell carcinoma dataset. Given the absence of annotated cell type labels in this dataset, we utilized two metrics, the SC and the CHI, to evaluate clustering performance. Since this dataset lacks histology images, SpaGCN and stLearn are not applicable. Therefore, the methods used for comparison include both multi-slice integration methods, such as STAligner and GraphST, as well as single-layer slice methods like Louvain, Leiden, SEDR and STAGATE.

**Figure 6:**
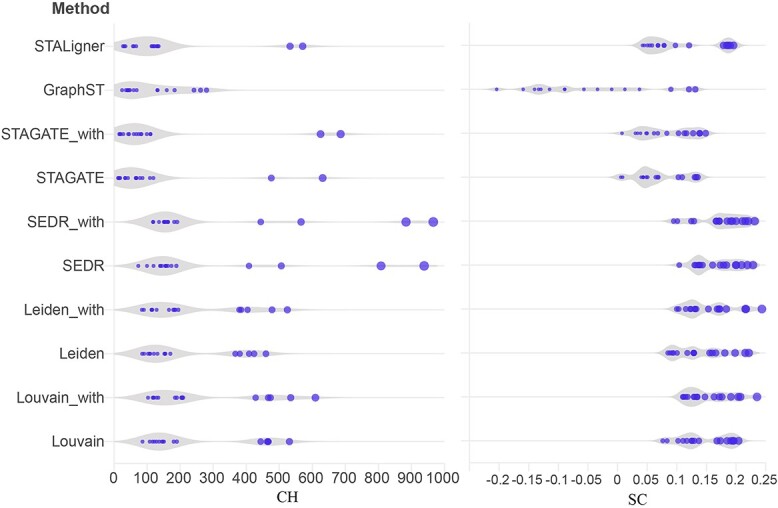
The SC and CHI metrics in the human skin squamous cell carcinoma dataset. The ‘_with’ suffix denotes methods that have been integrated with BiGATAE, such as ‘SEDR_with’, which signifies the SEDR method integrated with BiGATAE.

Figure 6 illustrates the clustering results of these methods associated with metrics for each slice. Two natural conclusions can be drawn from Figure 6: (1) methods integrated with BiGATAE consistently outperform their non-integrated counterparts, highlighting the effectiveness of BiGATAE. (2) On this dataset, methods specifically designed for multi-slice integration, such as STALigner and GraphST, do not exhibit superior clustering results compared to single-layer slice methods. GraphST, in particular, yields the lowest performance among all the methods. SEDR outperforms STALigner. Louvain, Leiden, STAGATE exhibit similar performance as STALigner. However, each method integrated with BiGATAE consistently surpasses its original version. Among them, SEDR_with represents the SEDR method integrated with BiGATAE, achieving the best performance, and Louvain_with and Leiden_with represent the Louvain and Leiden methods integrated with BiGATAE, delivering particularly promising results.

### BiGATAE improves the performance of Louvain and Leiden on the breast cancer dataset

**Figure 7:**
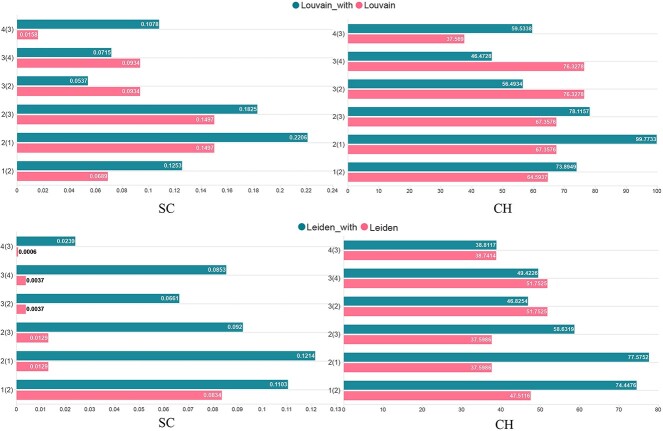
The SC and CHI metrics in the breast cancer dataset using Louvain and Leiden. In the parentheses, the number indicates the adjacent slice that provides information assistance. For example, ‘1(2)’ signifies that slice 1 is the target slice, and slice 2 is the adjacent slice. The ‘_with’ suffix denotes methods that have been integrated with BiGATAE.

This dataset consists of four consecutive slices, and the absence of some crucial parameters renders many methods inapplicable. On this dataset, our primary focus was to compare the performance of Louvain and Leiden before and after integrating BiGATAE, as shown in Figure 7. For the Leiden method, integrating BiGATAE significantly improves algorithm performance across the board. As for the Louvain method, in most cases, integrating BiGATAE results in improved algorithm performance.

## DISCUSSION

In this paper, we introduced the BiGATAE method, which facilitates the exchange of information between adjacent slices. It can be seamlessly integrated with existing single-slice methods, enhancing the accuracy of existing methods in identifying spatial domains and extending single-slice methods for the integration of multiple consecutive slices. BiGATAE first involves employing the PASTE method to establish correspondences between spots in adjacent slices, constructing a bipartite graph. Then it effectively transfers information between adjacent slices inspired by graph attention networks. Finally, it can seamlessly interface with existing single-slice clustering methods.

We conducted benchmarks on three datasets to showcase the capabilities of BiGATAE. The experimental results demonstrate that integrating BiGATAE significantly enhances the performance of single-layer slice clustering methods, underscoring the role of BiGATAE in boosting clustering from a global topological perspective. This enhancement is particularly prominent at the boundaries between different cell types. Furthermore, our results indicate that clustering methods integrated with BiGATAE outperform those explicitly designed for multi-slice integration.

The first step of the BiGATAE method is to establish correspondences between different spots in adjacent slices, and the accuracy of alignment between adjacent slices has a substantial impact on the BiGATAE method. In the future, we will consider the integration and transmission of information from multiple consecutive slices for global communication. A straightforward approach is to expand the adjacency matrix in Figure 1 to an $N\times (mN)$ dimension, where $N$ is the number of spots in a single slice, and $m$ is the number of slices to be integrated. Moreover, the adjacency matrix in Figure 1 can be upgraded from a binary matrix to a probability similarity matrix between two spots, accounting for the varying impact of spots from different slices on the target spot.

Additionally, the current BiGATAE method primarily conveys gene expression information between adjacent slices. With the increasing availability of histology images, we will investigate the transmission of both gene expression and histology image information across consecutive slices.

**Key Points**

- BiGATAE can be seamlessly integrated with existing single-slice methods, enhancing the accuracy of existing methods in identifying spatial domains and extending single-slice methods for the integration of multiple consecutive slices.
- BiGATAE is a bipartite graph attention auto-encoder.
- BiGATAE significantly enhances the performance of single-layer slice clustering methods, underscoring the role of BiGATAE in boosting clustering from a global topological perspective.
- Clustering methods integrated with BiGATAE outperform those explicitly designed for multi-slice integration.

## Data Availability

The ‘human dorsolateral prefrontal cortex dataset’ was downloaded from http://research.libd.org/spatialLIBD. ‘The breast cancer dataset’ was downloaded from the Dryad Digital Repos. The human skin squamous cell carcinoma dataset’ was downloaded from NCBI GEO under the accession number GSE144239.

## CODE AVAILABILITY

The implementation of BiGATAE is accessible through the GitHub repository at https://github.com/fdu-wangfeilab/BiGATAE.

## Author Biographies

**Tao Yuhao** is currently a PhD candidate at the School of Computer Science and Technology, Fudan University, China. His research interests include deep learning, bioinformatics and computational biology.

**Sun Xiaoang** is currently a master candidate at the School of Computer Science and Technology, Fudan University, China. His interests are machine learning and bioinformatics.

**Wang Fei** is an associate Professor of Fudan University. Her research interests include biological data analysis, systems biology and artificial intelligence.
